# A Comparison of the Effects of Aerobic and Intense Exercise on the Type 2 Diabetes Mellitus Risk Marker Adipokines, Adiponectin and Retinol Binding Protein-4

**DOI:** 10.1155/2014/358058

**Published:** 2014-01-12

**Authors:** Amy Phillips, Christian Cobbold

**Affiliations:** School of Biosciences, Cardiff University, Museum Avenue, Cardiff CF11 3AX, UK

## Abstract

With a more sedentary population comes growing rates of obesity and increased type 2 diabetes mellitus (T2DM) risk. Exercise generally induces positive changes in traditional T2DM risk markers such as lipids, glucose tolerance, and insulin sensitivity; however alterations in concentrations of many circulating cytokines and their respective receptors are also becoming apparent. These cytokines may be early-response health risk factors otherwise overlooked in traditional T2DM risk marker analysis. Plasma levels of two adipocyte-originating cytokines, adiponectin and retinol binding protein 4 (RBP-4), alter following exercise. Adiponectin has anti-inflammatory, anti-atherosclerotic, and anti-insulin resistance roles and its secretion increases with physical activity, whilst elevated RBP-4 leads to increased insulin resistance, and secretion decreases with increasing physical activity; thus these plasma adipokine levels alter favourably following exercise. Although current data are limited, they do suggest that the more intense the exercise, the greater the positive effect on plasma RBP-4 levels, whilst lower intensity aerobic exercise may positively improve adiponectin concentrations. Therefore short-duration, high intensity training may provide a time-efficient alternative to the recommended 150 min moderate aerobic exercise per week in providing positive changes in RBP-4 and other traditional T2DM risk markers and due to increased compliance give greater health benefits over the longer term.

## 1. Introduction

An increase in sedentary lifestyle has led to a rise in obesity, with over 60% of UK adults and 30% of UK children being overweight. Obesity gives rise to the increased prevalence of type 2 diabetes mellitus (T2DM) and cardiovascular disease (CVD), two of the leading causes of mortality and morbidity [1, 2]. In the UK there are 3.5 million diagnosed diabetes sufferers, and prevalence is projected to increase to 5 million by 2025; this needs to be addressed to reduce mortality and morbidity rates and minimise the economic costs of T2DM and related diseases [3]. A sedentary lifestyle has been shown to disrupt biochemical processes including the preservation of specific insulin-sensitive glucose transporters (GLUT4), which help maintain constant plasma-glucose levels [4], and the regulation of anti-inflammatory mediators [5]. The disruption of these processes can be observed by measuring specific T2DM risk factors, such as mass index (BMI), lipid profile, body fat index (BFI), glucose tolerance and insulin sensitivity tests, and plasma inflammatory markers [5–8].

One of the consequences of T2DM is the body moving into an inflammatory state, and increased concentrations of cytokines, interleukins, and macrophages, all of which have pro-inflammatory characteristics, have been seen at elevated levels in the blood of obese and T2DM patients [1]. Some of these inflammatory regulators, such as adiponectin and RBP-4, have become a point of interest for research due to their roles in altering insulin sensitivity and their fluctuating concentrations corresponding to the health of the subject [1, 9, 10].

## 2. Role of Exercise in T2DM Prevention

**Figure 1:**
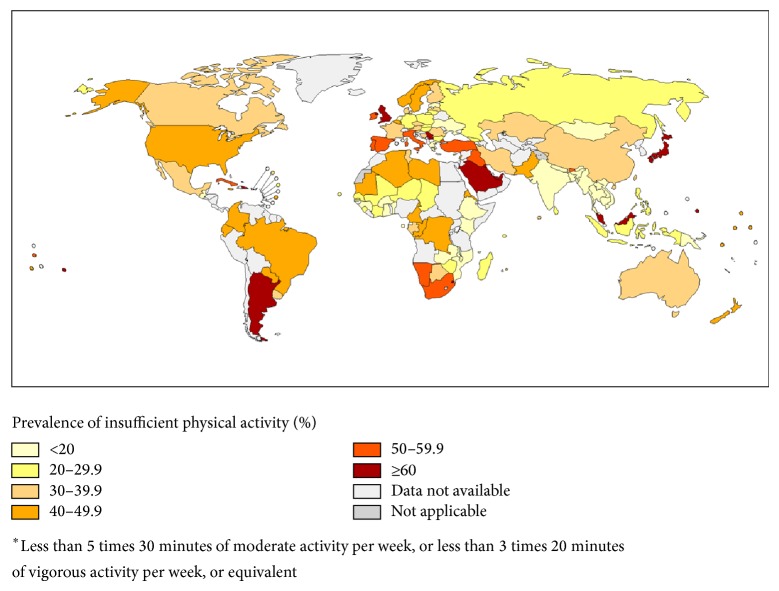
The Worldwide prevalence of adult physical inactivity. The prevalence of inactivity in the UK, highlighted in red, was 63% in 2008. Globally this value is approximately 31%. Taken from the Global Health Observatory of World Health Organization, 2008. The boundaries and names shown and the designations used on this map do not imply the expression of any opinion whatsoever on the part of the World Health Organization concerning the legal status of any country. Territory, city, or area or of its authorities, or concerning the delimitation of its frontiers or boundaries. Dotted lines on maps represent approximate border lines for which there may not yet be full agreement. Data source: World Health Organization. Map production: Public Health Information and Geographic Information System (GIS) World Health Organization. ©WHO 2011. All rights reserved.

Exercise is an important part of maintaining a healthy body. As well as aiding the maintenance of internal biochemical processes, exercise is an effective way of managing weight. Public health guidelines recommend individuals perform 150 minutes of moderate-intensity aerobic exercise a week in order to obtain optimal results, including weight loss, reduced blood pressure, and reduced cholesterol [8]. Despite the rewards, many people still complain that they “don't have the time” to exercise. Insufficient activity does not just affect the UK where approximately 65% of people do not undertake the recommended levels of exercise; it is a worldwide problem (Figure 1). Interestingly, research suggests that similar health benefits to 150 min/week aerobic exercise can be seen with 15 minute sessions of high-intensity interval training (HIIT), performed on alternating days [11, 12].

HIIT is described as repeated sessions of brief, intermittent “all-out” exercise, where subjects work at an intensity which is as close to their maximum VO_2max_ (aerobic capacity) as possible or greater [11]. A common HIIT session used by research scientists studying the effects of extreme exercise on the body is called a Wingate test. The Wingate test includes 30 seconds of maximum effort cycling, followed by 3-4 minutes of moderate effort cycling (see Table 1) [13]. This cycle is repeated 3–5 times, or until the subject is too exhausted to continue. The two main differences between HIIT and aerobic exercise are the intensity of the exercise and the time taken to complete one session of exercise. During a session of HIIT, individuals are subject to very high intensity exercise for a short period of time, sometimes as little as 3 minutes per week, reaching VO_2max_ levels of 90% or greater; during aerobic exercise, VO_2max_ levels only rise to about 60% effort. Whether HIIT produces comparable health benefits to current public health guidelines has been investigated in multiple studies [14–16].

The aim of this review is to provide an up-to-date analysis on the role of different exercise types on the levels of key adipokines adiponectin and RBP-4 and discuss how this may be used to help manage those with or at risk of T2DM.

## 3. Adiponectin

Adiponectin is a cytokine released from visceral adipocytes which is known to have anti-inflammatory, anti-atherosclerotic, and anti-insulin resistance characteristics [17, 18]. *In vivo* studies have indicated a negative association between plasma tumour necrosis *α* (TNF*α*) and adiponectin levels; increased TNF*α* secreted from adipocytes significantly suppresses adiponectin expression in a dose-dependent manner [19], and its secretion from adipocytes is therefore inversely correlated to the total lipid store and is inhibited by the accumulation of fat [1]. It has also been suggested that plasma adiponectin concentration may be the strongest and most consistent biochemical predictor of T2DM [20]. Patients diagnosed with T2DM, hypertension, and ischemic heart disease often have low adiponectin concentrations in the blood, which is now a recognised risk factor for CVD [21].

Several studies have looked at the effects of exercise on adiponectin levels. In an 8-week study, previously sedentary, overweight subjects (*n* = 24) performed the government recommended 150 minutes of aerobic exercise per week in the form of walking and bicycling [22]. Adiponectin concentration and waist circumference were measured at the start and the end of the study. It was found that, at the end of the trial, adiponectin plasma levels had significantly increased under the exercise plan from 11.9 mg/L to 12.5 mg/L (*P* < 0.05) and that adiponectin levels were inversely correlated to waist circumference; there was a concomitant significant drop in waist circumference. The study also found that there was a positive correlation between adiponectin levels and microvascular blood flow, indicating that an increase in adiponectin levels may reduce the risk of cardiovascular disease [22]. A similar 8-week aerobic training study in healthy middle age men (*n* = 15) also showed a significant increase in adiponectin levels following training, which correlated with a decrease in body fat percentage [18]. Whilst both of these studies had relatively small sample sizes, they do suggest that aerobic training leads to increased adiponectin levels and thus increased insulin sensitivity; importantly, both studies showed correlations between increased adiponectin levels and reduced levels of adiposity. Furthermore, Yu et al. looked at associations between physical activity and the relative concentrations of inflammatory markers [23] using a modified form of the International Physical Activity Questionnaire. It was found that, in those who regularly did exercise, the concentration of adiponectin was significantly higher than that in those who only occasionally did exercise.

Whilst there are several studies showing the positive benefits of aerobic exercise on adiponectin levels, evidence of the effects of HIIT on adiponectin is less clear. An article by Richards and colleagues in 2010 studied the effects of a 2-week programme of short-term sprint interval training, consisting of approximately six sessions of 4–7 attempts of maximal effort on a stationary bike [24]. All attempts lasted 30 seconds, with 4 minutes of recovery time in-between, with the six sessions separated by 1-2 days. The study showed that, while insulin sensitivity increased during the trial, there was no significant difference in plasma adiponectin levels between the start and end of the trial. The increase in insulin sensitivity observed is most likely down to increased GLUT4 expression in skeletal muscle following HIIT; it is thought that HIIT rapidly depletes glycogen stores, which activates glycogen synthase and enhances GLUT4 synthesis and translocation to the plasma membrane, thus increasing insulin sensitivity and glucose uptake, both of which serve to replenish glycogen stores [15]. In contrast, Blüher et al [25] showed a significant increase in adiponectin levels following 4-week intense exercise (but not HIIT) in the normal glucose tolerance, impaired glucose tolerance, and T2DM groups compared to baseline values. In each case there was also a significant drop in % fat mass, indicating this may be one of the mechanisms for the increase in adiponectin observed. Interestingly, the number of cell membrane adiponectin receptors also rose, suggesting an increase in adiponectin sensitivity.

Together, these data suggest that different forms of exercise may have varying effects on adiponectin levels, with HIIT potentially having a lesser effect than the more time-and energy-consuming aerobic exercise where fat content is more likely to be reduced. This indicates that depletion of fat mass may be more important in changing adiponectin levels than exercise intensity.

## 4. Retinol Binding Protein-4

Also released from adipocytes, retinol binding protein-4 (RBP-4) is associated with multiple functions in the body, including fibrosis, the transport of retinol (vitamin A) to the eye, and increased insulin resistance [26, 27]. RBP-4 increases insulin resistance by suppressing peripheral expression of GLUT4 transporters [1, 26]. The presence of RBP-4 leads to increased macrophage migration to the site of RBP-4 release in adipose tissue which causes cytokine release and increases the risk of inflammation and CVD [1].

As expected, studies have shown that circulating concentrations of RBP-4 and the expression of its corresponding mRNA in adipose tissue are higher in those diagnosed with T2DM and impaired glucose tolerance and in obese patients [9]. Elevated levels of RBP-4 are often seen before the onset of T2DM, and some research indicates that regions near the RBP-4 locus on the human 10q chromosome can be used for identifying those at risk of T2DM [26, 28].

Most studies have shown that RBP-4 levels fall following exercise [23, 26, 29], and the more intense the exercise, the greater the fall in RBP-4, with moderate to low intensity exercise having insignificant effects on the concentration of RBP-4 [23, 29]. Interestingly, in a 10-week study of 74 women of varying ages, the effects of exercise were greater in those individuals that had a higher initial RBP-4 circulating concentration: the women with the highest initial RBP-4 concentrations were those aged 50 years and above, presumably as body fat, and therefore RBP-4 concentrations tend to increase with age. It was found that plasma RBP-4 fell significantly from 48.1 *μ*g/L to 38.0 *μ*g/L in the older women and that fall was significantly greater than that of the younger women [30].

As previously stated, many studies of the effects of exercise on the levels of RBP-4 have concluded that the more intense the exercise, the greater the fall in RBP-4. A 12-week study researching the effects of resistive and aerobic exercise on the plasma concentrations of RBP-4 in individuals diagnosed with T2DM showed that the change in RBP-4 concentration was significantly greater (*P* < 0.05) in the group that performed resistance exercise (−16.4 *μ*g/mL) than the group that performed aerobic (walking) exercise (−2.3 *μ*g/mL), or the control group (no exercise) (1.2 *μ*g/mL) [29]. They also showed a positive correlation between the change in the plasma RBP-4 concentrations and concentration of mid-thigh subfascial adipose tissue in the resistance group, observing that as this fat decreased, so did the concentration of RBP-4; this effect was not seen in either the aerobic or control groups [29].

Despite evidence suggesting that a fall in plasma RBP-4 concentration evokes insulin sensitivity and that resistance training and HIIT provides better results than aerobic exercise [1, 9, 26] in positively changing RBP-4 levels, Ku et al. found that there was no significant change in insulin sensitivity in either the aerobic or resistance exercise groups [29]. Further examination of their raw data following the trial showed that those with better baseline insulin sensitivity tended to have been assigned to the aerobic training group, despite the allocation process being random. This may have affected their results, and more studies will need to be carried out to substantiate this and to discover if more intense exercise or resistance training does provide significantly greater changes in insulin sensitivity than aerobic exercise.

## 5. Conclusions

The aim of this report was to analyse the effects of exercise on the fluctuating concentrations of the adipokine inflammatory markers adipokines, adiponectin and RBP-4. There is a clear negative correlation between adiponectin and adipocyte concentrations, with an increase in the secretion of adiponectin produced by increased physical activity, presumably due to decreased fat levels [22, 23]. Increases in adiponectin can help to reduce risk factors of CVD by decreasing inflammation and increasing microvascular blood flow and insulin sensitivity [17, 18, 22].

Despite the lack of evidence linking the two, RBP-4 seems to act opposite to adiponectin, showing positive correlations between RBP-4 concentrations and adipocyte concentration [29, 30]. RBP-4 levels fall following exercise and the more intense or resistive the exercise, the greater the extent of the fall [23, 26, 29]. Increases in the concentration of RBP-4 were also shown to be linked with the increase of some risk factors of CVD, such as increased inflammation and insulin resistance [1, 9].

Therefore, whilst moderate aerobic exercise and HIIT show positive benefits in terms of overall T2DM risk, there are conflicting results with regard to their effects on adiponectin and RBP-4. Moderate aerobic exercise seems to have a greater positive effect on adiponectin than HIIT, whilst HIIT and resistive training may have an improved response on RBP-4 levels. It is important to point out, however, that few studies have compared the effects of HIIT, resistive training, and moderate aerobic exercise on circulating plasma adipokine levels and current information is inconclusive. Future intervention studies need to be designed to compare the effects of HIIT and moderate aerobic exercise on RBP-4 and adiponectin levels, as well as other risk markers of T2DM, such as lipid profile, insulin sensitivity, glucose tolerance, and aerobic capacity. This should allow improved management of those with, or at risk of, T2DM.

## Figures and Tables

**Table 1:** A conventional Wingate test. This is a basic template. Researchers will change the times, intensity, and mode of exercise in order to fit their particular trial.

Time (s)	Wingate test								
	210	30	210	30	210	30	210	30	210
Exercise intensity	Moderate	High	Moderate	High	Moderate	High	Moderate	High	Moderate
VO_2 max_	40–60%	90%	40–60%	90%	40–60%	90%	40–60%	90%	40–60%
Phase	Warm-up	Sprint 1	Rest 1	Sprint 2	Rest 2	Sprint 3	Rest 3	Sprint 4	Cool down

## References

[1] DeBoer M. D. (2013). Obesity, systemic inflammation, and increased risk for cardiovascular disease and diabetes among adolescents: a need for screening tools to target interventions. *Nutrition*.

[2] Wang Y. C., McPherson K., Marsh T., Gortmaker S. L., Brown M. (2011). Health and economic burden of the projected obesity trends in the USA and the UK. *The Lancet*.

[3] NHS (2013). National statistics: statistics on obesity, physical activity and diet: England, 2013.

[4] Kahn S. E., Hull R. L., Utzschneider K. M. (2006). Mechanisms linking obesity to insulin resistance and type 2 diabetes. *Nature*.

[5] Balducci S., Zanuso S., Nicolucci A. (2010). Anti-inflammatory effect of exercise training in subjects with type 2 diabetes and the metabolic syndrome is dependent on exercise modalities and independent of weight loss. *Nutrition, Metabolism and Cardiovascular Diseases*.

[6] O'Hagan C., de Vito G., Boreham C. A. (2013). Exercise prescription in the treatment of type 2 diabetes mellitus: current practices, existing guidelines and future directions. *Sports Medicine*.

[7] Balducci S., Zanuso S., Nicolucci A. (2010). Effect of an intensive exercise intervention strategy on modifiable cardiovascular risk factors in subjects with type 2 diabetes mellitus: a randomized controlled trial: the Italian Diabetes and Exercise Study (IDES). *Archives of Internal Medicine*.

[8] Bassuk S. S., Manson J. E. (2005). Epidemiological evidence for the role of physical activity in reducing risk of type 2 diabetes and cardiovascular disease. *Journal of Applied Physiology*.

[9] Graham T. E., Yang Q., Blüher M. (2006). Retinol-binding protein 4 and insulin resistance in lean, obese, and diabetic subjects. *The New England Journal of Medicine*.

[10] Højlund K., Frystyk J., Levin K., Flyvbjerg A., Wojtaszewski J. F. P., Beck-Nielsen H. (2006). Reduced plasma adiponectin concentrations may contribute to impaired insulin activation of glycogen synthase in skeletal muscle of patients with type 2 diabetes. *Diabetologia*.

[11] Gibala M. J., McGee S. L. (2008). Metabolic adaptations to short-term high-intensity interval training: a little pain for a lot of gain?. *Exercise and Sport Sciences Reviews*.

[12] Warburton D. E. R., McKenzie D. C., Haykowsky M. J. (2005). Effectiveness of high-intensity interval training for the rehabilitation of patients with coronary artery disease. *American Journal of Cardiology*.

[13] Gibala M. J., Little J. P. (2010). Just HIT it! A time-efficient exercise strategy to improve muscle insulin sensitivity. *Journal of Physiology*.

[14] Burgomaster K. A., Howarth K. R., Phillips S. M. (2008). Similar metabolic adaptations during exercise after low volume sprint interval and traditional endurance training in humans. *Journal of Physiology*.

[15] Gibala M. J., Little J. P., Macdonald M. J., Hawley J. A. (2012). Physiological adaptations to low-volume, high-intensity interval training in health and disease. *Journal of Physiology*.

[16] Tjønna A. E., Lee S. J., Rognmo Ø. (2008). Aerobic interval training versus continuous moderate exercise as a treatment for the metabolic syndrome: a pilot study. *Circulation*.

[17] Einvik G., Flyvbjerg A., Hrubos-Strøm H. (2013). Novel cardiovascular risk markers in depression: no association between depressive symptoms and osteoprotegerin or adiponectin in persons at high risk for sleep apnea. *Journal of Affective Disorders*.

[18] Rashidlamir A., Saadatnia A. (2012). The effect of eight weeks of aerobic training on the plasma level of adiponectin, leptin, and resistin in healthy middle-aged men. *Science & Sports*.

[19] Su H., Lau W. B., Ma X.-L. (2011). Hypoadiponectinaemia in diabetes mellitus type 2: molecular mechanisms and clinical significance. *Clinical and Experimental Pharmacology and Physiology*.

[20] Li S., Shin H. J., Ding E. L., van Dam R. M. (2009). Adiponectin levels and risk of type 2 diabetes: a systematic review and meta-analysis. *Journal of the American Medical Association*.

[21] Koenig W., Khuseyinova N., Baumert J., Meisinger C., Löwel H. (2006). Serum concentrations of adiponectin and risk of type 2 diabetes mellitus and coronary heart disease in apparently healthy middle-aged men: results from the 18-year follow-up of a large cohort from southern Germany. *Journal of the American College of Cardiology*.

[22] Pasqualini L., Schillaci G., Innocente S. (2010). Lifestyle intervention improves microvascular reactivity and increases serum adiponectin in overweight hypertensive patients. *Nutrition, Metabolism and Cardiovascular Diseases*.

[23] Yu Z., Ye X., Wang J. (2009). Associations of physical activity with inflammatory factors, adipocytokines, and metabolic syndrome in middle-aged and older chinese people. *Circulation*.

[24] Richards J. C., Johnson T. K., Kuzma J. N. (2010). Short-term sprint interval training increases insulin sensitivity in healthy adults but does not affect the thermogenic response to beta-adrenergic stimulation. *The Journal of Physiology*.

[25] Blüher M., Bullen J. W., Lee J. H. (2006). Circulating adiponectin and expression of adiponectin receptors in human skeletal muscle: associations with metabolic parameters and insulin resistance and regulation by physical training. *Journal of Clinical Endocrinology and Metabolism*.

[26] Christou G. A., Tselepis A. D., Kiortsis D. N. (2012). The metabolic role of retinol binding protein 4: an update. *Hormone and Metabolic Research*.

[27] Brogan A. P., Dickerson T. J., Boldt G. E., Janda K. D. (2005). Altered retinoid homeostasis catalyzed by a nicotine metabolite: implications in macular degeneration and normal development. *Proceedings of the National Academy of Sciences of the United States of America*.

[28] Duggirala R., Blangero J., Almasy L. (1999). Linkage of type 2 diabetes mellitus and of age at onset to a genetic location on chromosome 10q in Mexican Americans. *American Journal of Human Genetics*.

[29] Ku Y. H., Han K. A., Ahn H. (2010). Resistance exercise did not alter intramuscular adipose tissue but reduced retinol-binding protein-4 concentration in individuals with type 2 diabetes mellitus. *Journal of International Medical Research*.

[30] Lim S., Sung H. C., Jeong I.-K. (2008). Insulin-sensitizing effects of exercise on adiponectin and retinol-binding protein-4 concentrations in young and middle-aged women. *Journal of Clinical Endocrinology and Metabolism*.

